# Study of the Immune Response in the Elderly: Is It Necessary to Develop a Vaccine against *Neisseria meningitidis* for the Aged?

**DOI:** 10.1155/2019/9287121

**Published:** 2019-08-22

**Authors:** Gabriela Trzewikoswki de Lima, Elizabeth De Gaspari

**Affiliations:** ^1^Department of Immunology, Adolfo Lutz Institute, São Paulo, Brazil; ^2^Interunits Post-Graduate Program in Biotechnology, University of São Paulo, São Paulo, Brazil

## Abstract

Literature reports the association between aging and decline in the immune system function. The elderly have a higher risk of developing infectious diseases and are often less responsive to vaccines that are effective in the young. The case fatality rate of invasive meningococcal disease is higher in the elderly; therefore, vaccination for this population should be evaluated. Although new vaccines have been developed against *Neisseria meningitidis*, there is still a need to evaluate a vaccine for those older than 60 years, as the currently licensed vaccines are not indicated for this population.

## 1. Introduction

Immune responses are essential to promote the organism's defense. However, aging seems to be associated with the dysfunction of the immune response [1]. This process is called immunosenescence and is associated with increased susceptibility of the elderly to developing infections, cancer, and autoimmune diseases. Moreover, the responses to vaccines are usually reduced in the elderly [2].

Aging is associated with a paradox where a state of basal chronic inflammation, prevalent even in the absence of diseases, coexists with a state of immunodeficiency [3]. This persistent inflammation, commonly called inflammaging, is possibly associated with continuous exposure to antigens, combined with the increase in the secretion of proinflammatory cytokines by senescent cells and oxidative stress, which involves the production of free radicals and toxic products [4].

**Figure 1:**
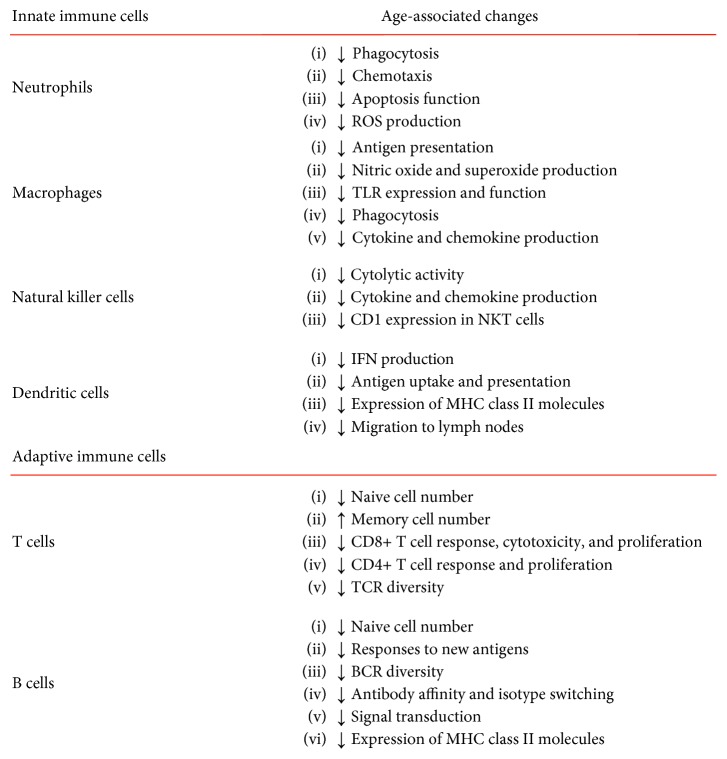
Changes associated with aging in cells of innate and adaptive immunity.

The process of aging affects both innate and adaptive immune responses; however, the first seems to be less affected [5].  Figure 1 shows the main changes associated with aging in cells of the innate and adaptive immune system.

The innate immune response is the body's first line of defense against pathogens. The innate immune cells recognize and react to pathogen-associated molecular patterns (PAMPs) and damage-associated molecular patterns (DAMPs) through specific receptors and play a role in eliminating invasive pathogens. Besides, the innate immune system possess physical barriers, such as the epidermis [6].

Collectively, the main characteristics of the aging process regarding the innate system are the immune stimulation in the basal level on the one hand and immune paralysis when specific functions are needed, such as phagocytosis, chemotaxis, presentation of antigens, and production of cytokines and reactive oxygen species (ROS), on the other hand [7]. The expression of pattern-recognition receptors (PRRs) and costimulatory molecules also seems to be decreased in the innate immune cells [8].

The adaptive immune system is composed of cellular and humoral immune responses. T cells are the main components of the cellular response and are basically divided into two populations, CD4+ and CD8+, according to their functions. CD4+ T cells are also called helper T cells and regulate the function of other cells of the immune system. CD8+ T cells, also called cytotoxic T cells, are responsible for eliminating infected cells. B lymphocytes have the main function of producing antibodies and, therefore, are related to the humoral immune response. The adaptive immune response depends on the generation of a vast repertoire of antigen receptors in these cells and their subsequent activation and clonal expansion. The activation of the adaptive immune response depends not only on the recognition of antigens but also on secondary signs provided by the innate immune response [6].

Thymic involution is one of the most prominent characteristics of aging and is associated with the decline of naive T cells. During the last decades, it has become increasingly clear that, along the adulthood, the homeostasis of T cells is maintained primarily by the peripheral proliferation of naive and memory T cells and not by the production of new T cells [9].

When evaluated separately, T cell subsets show a reduction of two- to fivefold in the number of naive T cells in healthy elderly individuals [10]. Also, naive T cells of the elderly present decreased cytokine production, less clonal expansion, and decreased expression of activation markers after the primary antigen presentation by antigen-presenting cells (APCs) [11].

Despite that the number of effector and memory T cells increases with age, the vaccination response in the elderly is impaired, which is indicative of the gradual decline of functional response [12]. In fact, it is observed that senescent T cells present defects in activation, memory, signaling, clonal expansion, and development of antigen-specific effector cells and long-lived memory cells [5].

As cytokines modulate the immune response, it has been proposed that changes in the cytokine production may contribute to the functional defects of T cells. With aging, there is a change in the cytokine profile, for predominantly IL-4 and IL-10 [13].

Furthermore, the repertoire of T-cell receptors (TCRs) present in individuals between 70 and 85 years is significantly smaller than the one found in individuals between 20 and 35 years [10]. Senescent T lymphocytes also present reduced expression of cell surface receptors, such as CD28 (important in lymphocyte activation) and CD27 (associated with the proliferative capacity of T lymphocytes) [14, 15]. The expression of the costimulatory molecule CD40L can also be reduced in CD4+ T cells of elderly individuals, which can affect the response in the germinal centers [16].

The number of B-cell precursors in the bone marrow of the elderly remains relatively stable, but there is a significant reduction in the number of mature B cells in the peripheral blood [17]. Also, there are a limited diversity of B-cell receptors (BCRs) and a decrease in the population of naive B cells. The intrinsic defects of B cells related to aging include a decrease in the expression of activation-induced cytidine deaminase (AID) and a decreased number of switched memory B cells [1]. This leads to the production of short-lived, low-affinity antibodies that may have defects in isotype switching, reflecting a low ability to respond effectively against pathogens [11].

## 2. Vaccination in the Elderly

The decrease in birth rate and increase in life expectancy have caused the progressive rise of the elderly population worldwide [5]. The increasing population density of those aged more than 65 years requires new strategies to ensure that health and well-being remain with advancing age.

Infections are one of the leading causes of morbidity and mortality in the elderly and may present different clinical features of those observed in young adults, regarding signs, symptoms, and progression, which can hinder the early diagnosis and impair the treatment, making their prevention even more important [5].

The susceptibility to infections, such as influenza virus, meningococcus, group B streptococcus, pneumococcus, respiratory syncytial virus, and varicella-zoster virus, becomes higher in this age group. As such, they need more frequent booster vaccinations, in many cases with vaccines specifically designed to stimulate the immune system of the elderly to respond better to vaccination [18]. Therefore, vaccinating older people with existing vaccines or developing new improved vaccines against pathogens that affect this population is one of the main interventions to prevent infections and ensure the health of the elderly.

Few vaccines are recommended to the elderly, such as influenza, herpes zoster, diphtheria, tetanus, pertussis, hepatitis, and pneumococcal vaccines [11]; however, these vaccines are less effective in the aged population than in the young [19].

The Centers for Disease Control and Prevention (CDC) estimates that the efficacy of the influenza vaccine in those aged less than 65 years is 70–90%, whereas in those aged 65 years or older, the efficacy is 30–40% [20]. Likewise, the efficacy of the attenuated virus vaccine against herpes zoster (ZOSTAVAX®) decreases with aging: 69.8% in individuals between 50 and 59 years, 64% in people between 60 and 69 years, 41% in people between 70 and 79 years, and only 18% in individuals above 80 years [21].

The most common approaches to improve the effectiveness of vaccines in the elderly include increasing the antigenic content per dose, changing the route of administration, giving booster doses, and using adjuvants [22]. The use of adjuvants has the aim to potentialize activation of APCs, cytokine production, and stimulation of B and T cells. Different routes of administration may deliver the antigen to sites enriched with APCs, optimizing its activation and presentation of antigens. The increase in the antigenic dose has the purpose to improve their presentation and, therefore, the activation of T cells [23].

Several strategies to improve the effectiveness of influenza vaccines in the elderly were addressed successfully, such as the use of adjuvant MF59 [24], the increase in the antigenic content from 15 *μ*g to 60 *μ*g per dose [25], and a vaccine administered via the intradermal route rather than the intramuscular route [26]. These vaccines stimulated a higher humoral response compared to those previously used, especially the MF59-adjuvanted vaccine. A study conducted in Italy showed that the risk of hospitalization for influenza or pneumonia was 25% lower in subjects vaccinated with the adjuvanted vaccine than in those vaccinated with the nonadjuvanted vaccine [27]. Another example is the novel herpes zoster subunit vaccine, which contains the varicella-zoster virus E glycoproteins and the AS01B adjuvant, yielding 97.9% efficacy, regardless of age, unlike the vaccine ZOSTAVAX® mentioned above, which presents reduced efficacy in the elderly [28].

## 3. Meningococcal Vaccines

Meningococcal disease can progress very rapidly, and death can occur within 24 to 48 hours after the initial symptoms. The response of B cells to meningococcal infection may not occur quickly enough since immune memory will mount an adequate defense against a known antigen in approximately 2 to 7 days, and the incubation period of meningococcal disease is 3 to 4 days. Therefore, the maintenance of protective levels of circulating antibodies by vaccination is important in prevention against meningococcal disease [29].

Meningococcal vaccines produced from capsular polysaccharides were introduced in the 70s decade; however, they are poorly immunogenic in toddlers and do not induce immunologic memory. These vaccines induce mainly IgM and short-lived bactericidal antibodies. Besides, repeated immunization with this vaccine may induce hyporesponsiveness [30, 31]. The conjugation of the capsular polysaccharide to a carrier protein improves its immunogenicity because it allows a T-cell-dependent response, inducing high-avidity antibodies, higher bactericidal activity, immunologic memory, and responsiveness to booster doses [32]. Nowadays, polysaccharides and conjugated vaccines against serogroups A, C, W-135, and Y are available [33]. Polysaccharide and conjugated vaccines to serogroup B were developed; however, these vaccines did not induce an effective humoral response [34, 35], and this might be due to the structural similarity of the capsular polysialic acid *α*2 ⟶ 8 and the embryonic neural cell adhesion molecules (N-CAMs) [36]. This similarity is a concern as it might lead to the development of autoimmunity. It has been reported the presence of IgM antibodies is directed against embryonic N-CAMs in the serum of patients suffering from group B meningitis [37]. The major concern in the administration of this vaccine would be the risk in women who become pregnant; if IgG antibodies against embryonic N-CAMs were developed after vaccination, there is a chance that these antibodies cross the placenta and cause damage to the fetal central nervous system [38]. Thus, many researchers concluded that developing a MenB polysaccharide vaccine presented more risks than benefits. So, MenB vaccine development was focused on subcapsular antigens, such as outer membrane proteins (OMPs) and outer membrane vesicles (OMVs) [39]. 4CMenB (Bexsero®) and rLP2086 (Trumenba®) are recently developed MenB vaccines, composed by OMPs; they are licensed in a few countries, and the initial studies suggest good immunogenicity and safety [30].

In the United States, between 1998 and 2007, 14.4% of invasive meningococcal disease (IMD) cases occurred in individuals aged 65 years or more; at that same period, the case fatality rate (CFR) of this age group was 23.2%, the highest between any age groups [40]. Also, in the United States, between 2006 and 2015, IMD cases in subjects aged >65 years accounted for 17.3% of total cases, and the CFR in those older than 85 years was higher than in any other age groups [41]. In Australia, in 2015, 20.1% of laboratory-confirmed cases of IMD occurred in people aged ≥65 years [42]. In Japan, between 2013 and 2014, most cases of IMD occurred in adults older than 50 years [43]. Approximately 14.7% of IMD cases occurred in individuals older than 50 years in an analysis of 25 European countries between 2004 and 2014 [44]. In France, between 2006 and 2015, 11.3% of IMD cases occurred in people aged 60 years or more, besides this age group presented the highest CFR [45]. Despite that the incidence of IMD in the elderly is relatively low, the CFR is high, so we consider it is important to evaluate the use of meningococcal vaccines to prevent disease in the elderly.

Table 1 presents characteristics of currently licensed meningococcal conjugate and protein vaccines, and these vaccines are not indicated to individuals over 55 years of age, given the lack of studies. The only meningococcal vaccine licensed to subjects older than 55 years is the plain polysaccharide ACWY (Menomune®) [56]. In some countries, meningococcal vaccines are indicated for individuals over 55 years considered at high risk (those with certain medical, occupational, or lifestyle indications and travelers to areas with high endemic rates for the infection) [57].

Dbaibo et al. [58] evaluated the immunogenicity and safety of a meningococcal conjugate ACWY-TT and a polysaccharide ACWY vaccine in adults over 55 years. After a single dose of immunization, ≥93.2% of the individuals that received the conjugated vaccine and ≥93.9% of the individuals that received the polysaccharide vaccine presented serum bactericidal antibodies (rSBA) with titers ≥1 : 128. The individuals over 65 years exhibited vaccine responses lower than those aged 56–65 years. Given these results, they concluded that these vaccines were immunogenic in the individuals evaluated.

Stamboulian et al. [56] assessed the immunogenicity of a meningococcal conjugate ACWY-CRM197 and a polysaccharide ACWY vaccine in individuals aged 56–65 years. The conjugate vaccine was considered superior to the polysaccharide one, achieving a higher percentage of seroresponse for all serogroups. The immunogenicity of MenACWY-CRM was similar between the groups aged 19–55 and 56–65 years.

Hutchins et al. [59] described that elderly individuals show decreased levels of antibodies after immunization with the meningococcal ACWY polysaccharide vaccine than young subjects and that the level of these antibodies decreased more rapidly. Besides, bactericidal activity in those aged 60–88 years was significantly lower.

Lalwani et al. [60] assessed the immunogenicity of a meningococcal ACWY-CRM conjugate vaccine in healthy Indian subjects aged 2 to 75 years and concluded that it generated a robust immune response; however, the aged subjects were included in a group of people aged 19–75 years, and so it was difficult to distinguish the specific response of the subjects older than 60 years in this study. The same occurred in the study of Ramasamy et al. [61], which compared the immunogenicity of a quadrivalent conjugate vaccine (MenACWY-CRM) with that of a quadrivalent polysaccharide vaccine (MenACWY-PS) in healthy adults aged 18–70 years; both vaccines were considered immunogenic, but again the older individuals were not evaluated separately.

The few studies about the effectiveness of meningococcal vaccines in the elderly [56, 58] suggest that it would be possible to adapt the currently available conjugate vaccines to older individuals, which would be more viable than developing new vaccines specifically for the elderly. As described earlier in this article, some vaccines, such as FLUAD®, Fluzone High-Dose®, Intanza® 15 *μ*g (influenza), and Shingrix® (varicella-zoster virus), have been specifically designed to improve the elderly's immune response. However, because of the relatively low incidence of meningococcal disease, it might not be interesting for the pharmaceutical industry to develop a new meningococcal vaccine targeting specifically the elderly.

The approach of expanding age indication to the already existing vaccines was used for the tetanus toxoid, diphtheria toxoid, and acellular pertussis (Tdap) and pneumococcal vaccines after studies indicated they were safe, well tolerated, and immunogenic in adults aged 65 years and older [62–65]. Furthermore, studies are being conducted to try to expand the age indication of other existing vaccines, such as the rotavirus [66].

Further studies are required to ensure the safety and efficacy of meningococcal vaccines in adults aged 65 years and older, as it has been described several changes in the immune system of the elderly may lead to diminished vaccine response. Furthermore, to our knowledge, there are no studies of meningococcal serogroup B vaccines such as 4CMenB (Bexsero®) and rLP2086 (Trumenba®) in individuals of this age group.

Perhaps, the vaccination of the elderly against *N. meningitidis* is not considered a priority because of the low incidence of the disease. Nevertheless, we believe that given the increasing proportion of older people in the population and the high CFR of meningococcal disease in the elderly, it would be interesting to evaluate the insertion of these vaccines in the immunization programs for this age group, especially in countries with high and intermediate endemic disease and during outbreaks. Also, vaccines can generate other benefits, such as a lower overall cost of healthcare.

Studies are required to evaluate if introducing meningococcal vaccines for adults aged 65 years and older into immunization programs is cost-effective, and in this way, health authorities can decide whether the benefit of vaccinating the elderly with these vaccines is worthwhile. Meningococcal disease is one of the several infections that can affect the elderly. Further studies about the burden of the infectious diseases in the elderly are necessary to define public health priorities and assess the need for vaccination against these pathogens.

Given the fact that the elderly are more likely to evolve to death when infected with *N. meningitidis* and the lack of studies of the meningococcal vaccines in the aged, we see a need to evaluate the current vaccines to assess their efficacy in this aged population and, if necessary, develop vaccines that are effective for them.

## Figures and Tables

**Table 1:** Meningococcal conjugate and protein vaccines currently licensed and their composition, age indication, and immunization schemes.

Vaccine	Pharma	Composition	Age group indicated	Immunization scheme	Reference
Menjugate®	GlaxoSmithKline (GSK)	Meningococcal C oligosaccharide conjugated with CRM197 + aluminum hydroxide	>2 months of age, teenagers, and adults	2–12 months: two doses with an interval of at least 2 months between the doses >12 months, teenagers, and adults: a single dose	[46]
Menactra®	Sanofi Pasteur	Meningococcal A, C, Y, and W-135 polysaccharides conjugated with DT	9 months through 55 years of age	9–23 months: two doses with an interval of at least 3 months between the doses 2–55 years: a single dose	[47]
MenAfriVac®	Serum Institute of India	Meningococcal A polysaccharide conjugated with TT + aluminum phosphate	1 year of age, adolescents, and adults up to 29 years of age	A single dose	[48]
Meningitec®	Pfizer	Meningococcal C oligosaccharide conjugated with CRM197 + aluminum phosphate	>6 weeks of age, adolescents, and adults	<12 months: three doses with an interval of at least 1 month between the doses >12 months, adolescents, and adults: a single dose	[49]
Meninvact®	Sanofi Pasteur	Meningococcal C oligosaccharide conjugated with CRM197 + aluminum hydroxide	>2 months of age, teenagers, and adults	2–12 months: two doses with an interval of at least 2 months between the doses >12 months, teenagers, and adults: a single dose	[50]
Menveo®	GlaxoSmithKline (GSK)	Meningococcal A, C, Y, and W-135 oligosaccharides conjugated with CRM197	>2 months through 55 years of age	2–6 months: four doses, administrated at 2, 4, 6, and 12 months of age 7–23 months, nonvaccinated: two doses with an interval of at least 3 months between the doses >2 years, teenagers, and adults through 55 years: a single dose	[51]
Neisvac-C®	Baxter	Meningococcal C polysaccharide conjugated with TT + aluminum hydroxide	>2 months and adults	2–12 months: two doses with an interval of at least 2 months between the doses >12 months, teenagers, and adults: a single dose	[52]
Nimenrix®	Pfizer	Meningococcal A, C, Y, and W-135 polysaccharides conjugated with TT	>12 months and adults	A single dose	[53]
Bexsero®	GlaxoSmithKline (GSK)	NHBA + NadA + fHbp + OMVs from NZ98/254 + aluminum hydroxide	>2 months and adults	2–5 months: three doses with an interval not less than 1 month; booster dose between 12 and 15 months 3-5 months: two doses with an interval of at least 2 months between the doses; booster dose between 12 and 15 months 6–11 months: two doses with an interval of at least 2 months between the doses; booster dose in the second year of life 12–23 months: two doses with an interval of at least 2 months between the doses; booster dose with an interval of 12 to 23 months between primary series 2 years and adults: two doses with an interval of at least 1 month between the doses	[54]
Trumenba®	Wyeth Pharmaceuticals	fHbp + aluminum phosphate	10 to 25 years of age	Three-dose schedule: 0, 1–2, and 6 months Two-dose schedule: 0 and 6 months	[55]

CRM197: *Corynebacterium diphtheriae* cross-reactive material 197; DT: diphtheria toxoid; fHbp: factor H binding protein; NadA: *Neisseria* adhesin A; NHBA: Neisserial heparin binding antigen; OMVs: outer membrane vesicles; TT: tetanus toxoid.
